# Towards a digital twin for smart resilient cities: real-time fire and smoke tracking and prediction platform for community awareness (FireCom)

**DOI:** 10.1007/s43762-025-00212-x

**Published:** 2025-10-11

**Authors:** Kijin Seong, Junfeng Jiao, Ryan Hardesty Lewis, Arya Farahi, Paul Navrátil, Nate Casebeer, Braniff Davis, Justice Jones, Dev Niyogi

**Affiliations:** 1 Urban Information Lab, The School of Architecture, The University of Texas at Austin, Austin, TX USA; 2 Project Lead, The Good Systems for Smart Cities, Austin, USA; 3 Cornell Tech, New York, NY USA; 4 Department of Statistics and Data Sciences, The University of Texas at Austin, Austin, TX USA; 5 Texas Advanced Computing Center, The University of Texas at Austin, Austin, TX USA; 6 Homeland Security Emergency Management, Austin, TX USA; 7 Austin Fire Department, Austin, TX USA; 8 Department of Geological Sciences, Jackson School of Geosciences, Department of Civil, Environmental and Architectural Engineering, and the Oden Institute for Computational Engineering and Sciences, The University of Texas at Austin, Austin, TX USA

**Keywords:** 3D digital twin, Smart city, Urban fire, Smoke prediction, Data aggregation

## Abstract

This paper discusses the development and application of a digital twin (DT) for urban resilience, focusing on an integrated platform for real-time fire and smoke. The proposed platform, FireCom, adapts DT concepts for the unique challenges of urban fire management, which differ significantly from regional wildfire systems. Through an exploratory case study in Austin, Texas, in the United States, this research bridges the theoretical foundations of 3D DT with their practical application in fire and smoke management. By fusing diverse data sources, ranging from air quality sensors and meteorological data to 3D urban infrastructure, FireCom supports both emergency response and public awareness through a publicly accessible dashboard. Unlike platforms developed primarily for wildland fire applications, FireCom is specifically designed to account for urban complexities such as building canyon effects on smoke dispersion and the heightened exposure risks associated with dense populations. This study contributes a scalable, replicable framework for municipalities seeking data-driven tools for proactive disaster management, with implications for broader climate resilience planning in urban areas.

## Introduction

Fire hazards seriously threaten the urban regions' functionality, particularly in highly populated metropolitan regions where city infrastructure systems are stressed (Shuman et al., 2022). In recent years, fire loss has grown rapidly among different communities, indicating that the average loss per structure fire in 2021 was $26,200, which is 1.5 times larger than the average loss in 1980 ($17,000) (Hall & Evarts, 2022). A stark illustration of this growing threat is the 2025 Palisades Fire in Los Angeles, which ignited on January 7 amid powerful Santa Ana winds. Over 24 days, the fire scorched approximately 23,448 acres, destroyed 6,837 structures, and resulted in 12 confirmed fatalities, marking it as the most destructive wildfire in Los Angeles city history and the third-most destructive in California's history (Cal Fire, 2025). Occurring at the wildland–urban interface, this event exposed a critical gap in existing wildfire monitoring systems: they are not equipped to model the complex interactions between fire, smoke, and the built environment. As a result, current platforms often fail to predict how smoke will channel through urban street canyons or to assess risks to critical infrastructure and vulnerable populations in real time. Addressing these limitations requires a city-scale DT capable of integrating high-resolution urban morphology, dense sensor networks, and predictive dispersion modeling to provide accurate, actionable insights for emergency management. Moreover, smoke is one of the leading causes of air pollution as it increases ambient fine particulate matter (PM2.5) concentration, threatening public health and safety (Horsley et al., 2018). In the urban context, the concept of resilient cities emerges as a crucial paradigm. Resilient cities are designed to absorb adversities, adapt to changing scenarios, and swiftly recover from disruptions (Walker et al., 2004), making them more equipped to handle challenges like urban fires. By harnessing state-of-the-art technologies, cities not only enhance their resilience against unpredictable events but also elevate their operations and service delivery. In line with this, the community’s co-produced real-time fire detection and smoke prediction, empowered by information and communications technologies (ICT) and machine learning, is gaining traction for its potential in pinpointing and overseeing fire and smoke events in urban settings (Bixler et al., 2022; Neirotti et al., 2014).

Recently, smart cities, artificial intelligence (AI), data analytics, and machine learning have contributed to a wealth of information that can be used to build a digital twin (DT) of a physical entity that can adapt to its environment (Kaur et al., 2020; White et al., 2021). DT allows the simulation of multiple options for optimized decision-making as an application in the real world. The concept of DT was first defined by the National Aeronautics and Space Administration (NASA) as “an integrated multiphysics, multiscale, probabilistic simulation of an as-built vehicle or system that uses the best available physical models, sensor updates, fleet history, etc., to mirror the life of its corresponding flying twin (Fuller et al., 2020; Glaessgen & Stargel, 2012).” The concept was initially adapted to the manufacturing domain, where simulation techniques were employed to create precise models of each component (White et al., 2021). This approach not only promoted efficiency but also facilitated electricity savings (Mendi, 2022). Recently, other sectors, including the military, as indicated by Mendi et al. (2022), have begun exploring its possible applications. When its concept is adapted to the area of urban planning, which is inherently multidisciplinary, the definition of DT is expanded to a digital representation of a socio-physical environment, which is updated and visualized to develop fast simulations of the present and future conditions in planning and implementation contexts, hence improving decision-making for practitioners and policymakers in the real world (El Saddik, 2018; Vrabič et al., 2018).

Research on a DT framework for fire and smoke management has received some attention recently. Studies considered how machine learning optimization uses integrated data fusion from multiple sources – satellites, Unmanned Aerial Vehicles (UAV), in-situ sensors, and social media – to enhance fire propagation simulations (Zohdi, 2020) and improve emergency response efficiency (Sadrabadi et al., 2025; Zohdi, 2020). As Zohdi (2021) specifically presented the application of aerial fire-fighting simulation in his following study, these algorithms can be generally utilized for fire-fighting and safe training for first responders (Zohdi, 2021). Meanwhile, research has also been underway to identify the limitations of the static environment of the fire spread algorithm, which is based on historical fire incident data or input data from users and to develop a dynamic fire spread algorithm using real-time data (Kim et al., 2019). In contrast to static conditions, real fire settings allow to generate unexpected wildfire spread findings due to frequent weather fluctuations and diverse fuel models (Cardil et al., 2019). Despite the benefit of using real-time data, little research has demonstrated the application of the data into the DT environments. Specifically, limited studies have investigated geographical and temporal integration to map fire detection and smoke prediction despite its broad impacts on community safety. Moreover, the geolocation of fire risks remains at the level of two-dimensional mapping, as the fire department’s safety monitoring systems have not been integrated with the 3D layouts of the urban system, leaving fire risks inaccurately portrayed.

To solve these issues, the development of fire-related digital twins has received growing attention from both academia and industry in recent years, reflecting the urgent need for predictive and integrated hazard management tools. Projects such as NASA’s Wildfire Digital Twin (NASA Science, 2024) and commercial platforms like OroraTech (Chesnokova, 2025) have demonstrated the power of combining real-time data, simulation, and AI to support wildfire management at scale. These advancements underscore the timeliness of our work. However, few existing systems are tailored for urban contexts or co-developed with municipalities to support community-scale risk communication.

Building on this foundation, we introduced the “FireCom” platform in the context of Austin, Texas, which is a city facing increasing risks from wildland-urban interface fires. FireCom is a real-time fire and smoke tracking system designed to enhance situational awareness and support emergency response. Developed in collaboration with the City of Austin, the platform is publicly accessible through an online dashboard to assist the Austin Fire Department and inform local communities. The framework integrates live fire incident data with predictive modeling to visualize potential smoke dispersion, enabling real-time monitoring and possible if–then scenario analysis for improved preparedness and communication. This web-based platform service is being designed not only to share the knowledge with diverse stakeholders effectively but also to help city officials warn schools, businesses, and neighborhoods of the risk of increasing air pollution and potential fire in the city and help them provide timely civic services based on real-time monitoring and prediction system.

While cyber-physical systems and the Internet of Things (IoT) are primarily designed for device interconnectivity and data exchange, DTs offer a holistic view of the systems, integrating real-time monitoring with predictive modeling and enabling a proactive approach to problems. In the context of the fire and smoke tracking and prediction platform, the advantages of employing DT become evident. Firstly, the DT’s innate capability for real-time monitoring aligns with our platform’s fire incident tracking, ensuring immediate and targeted responses. Secondly, DTs excel in predictive analysis, mirrored in our platform’s advanced 3D smoke prediction model, which simulates potential smoke spread, allowing for pre-emptive measures. The ability of DTs to perform integrated data fusion is reflected in our platform’s seamless assimilation of varied data sources like weather information, fire incidents, and air quality metrics into a cohesive unit. Lastly, our platform’s intuitive 2D and 3D visualizations reflect the user-friendly visualization that DTs promote, granting both fire professionals and the public a clear, unambiguous insight into the fire risks and smoke forecasts.

Within this context, the study presents a methodology grounded entirely in open data sources, allowing fire and smoke management strategies to be adapted and replicated across diverse cities and regions. It further demonstrates the integration of heterogeneous data streams into a unified model to enhance clarity and efficiency in analysis. The resulting platform, “FireCom,” offers layered 2D and 3D visualizations that empower both professionals and community members to interpret fire risks and smoke predictions in ways tailored to their needs. This work builds on the DT framework discussed in prior literature, applies the architecture to fire and smoke management, and outlines the building of a 2D and 3D web-based fire and smoke tracking platform and finally discusses opportunities and challenges in the fire/smoke DT for future research.

## Digital twin architecture for smart cities

### Framework of digital twin architecture

DT is developed as a simulation technique that incorporates multidisciplinary domains, physical quantities, multiple scales of sources, and the ability to develop probable outcomes through fast simulations. DTs have evolved as an integration of various cutting-edge technologies, including current IoT sensors, 5G connectivity, cloud platforms, large-scale data fusion, and artificial intelligence (Kaginalkar et al., 2021; Pan et al., 2020). Generally, access to large data resources or the ability to create large data through simulations becomes a basis for designing a virtual model or emulator, establishing and mapping a link between a digital environment and a physical entity.

Three components explain the DT characteristics: full life cycle, real-time or near-real-time, and bidirectional (Pan et al., 2020). First, applying to the urban system, the “full life cycle” implies that the DT is capable of managing the entire cycle of urban planning, including design, development, implementation, service, maintenance, and feedback. That is, it is not confined to assisting local governments in improving their services but also helping citizens improve their urban environments in daily life. Second, “real-time and near-real-time” denotes that a comprehensive connection based on a real-time link could be established between the ontology and DT. The real-time nature is the crucial component of the DT algorithms that makes the system linked cohesively. Finally, “bidirectional” indicates that information may go back and forth between the ontology and DT in both directions, making its relationship bidirectional and achieving co-production. The ontology sends data output to DT, while DT sends feedback information to the ontology. Within this iterative optimization and dynamic update process between the ontology and DT, the stakeholders can seek further interventions on the ontology based on the feedback from the DT.

To provide timely civic services and enhance communication between stakeholders, we propose an ontology-based model designed to integrate information collected from diverse sources. In this work, we refer to converting complex real-time/near-real-time information to explicit, machine-processable-type language as “ontology,” and we integrate these into a fire DT to enable a bidirectional feedback process between the physical environment and the virtual model. In FireCom, the ontology layer is tailored specifically for urban fire and smoke management, differing from generic smart-city DTs that focus on broad asset categories. Our ontology explicitly defines entities and relationships unique to fire-response contexts, including incident location and type, plume dispersion fields, environmental sensor streams, fire department coverage zones, and population vulnerability indices to fire. This domain-specific structuring enables the DT to map real-time sensor readings and predictive dispersion outputs directly to at-risk geographic units and operational resources. Bidirectional feedback in FireCom is operationalized through continuous updates: for instance, a detected PM2.5 spike in the sensor network could trigger a DT update to the smoke trajectory model, which might then update ontology tags for high-risk areas, potentially prompting adjusted resource allocation by fire departments.

Prior literature regarding DT modeling has established the architecture and framework. Data sources, data integration, and data sharing methods have been dynamically used to achieve semantic integration. Table 1 discusses general digital twin concepts and the architecture of the framework that addresses the application methods of fire emergency response and urban emergency management systems.

**Table 1:** Studies related to the architecture of the DT framework for fire hazard management

Study	DT Focus	DT framework	Data used for DT
General Digital Twin Implementation			
Mohammadi and Taylor (2017)	Smart city digital twin infrastructure analysis	VR-based analytic platform; AR-based mobile application; social media platform	Real-world social and sensor data; health-related data; city data
Zheng et al. (2019)	Application framework of digital twin	physical space; information processing layer; virtual space	Production data; equipment data; user data; service data; environment data
Pan et al. (2020)	Basic concepts of DT and applications	Physical layer; model layer; information layer; application layer	Physical space data, device data, environmental data
White et al. (2021)	Smart city DT for citizen feedback	Terrain layer; building layer; infrastructure layer; mobility layer; digital layer/smart city; virtual layer/digital twin	3D building data; infrastructure data; urban mobility and traffic data; urban IoT data
Jeong et al. (2022)	DT implementation with technology elements	Digital visualization; DT synchronization; modeling & simulation; federated DT; intelligent DT services	Multi-format (e.g., text, table, photo, and video) data; heterogeneous complex data; sensor data
Li et al. (2022)	Big data analysis of IoT in DT-based on deep learning	Service application layer; integration component layer; algorithm model layer; platform architecture layer; data absorbing layer; digital twin (physical vs. virtual space)	Multi-sourced big data; historical data (e.g., environmental data, water resources data, financial data, government data, business data, transportation data, etc.)
Kamath et al. (2024)	Building height data for DT urban modeling	Scalable city-wide 3D modeling	Building footprint and LiDAR-based height data
Razavi et al. (2024)	DT and Generative AI in smart city disaster planning	Conceptual architecture; AI-enhanced forecasting	Multisource data; simulation modeling
Yan et al. (2025a)	Common Data Environment (CDE) for scalable DT	CDE architecture; integration from building to city	BIM, GIS, real-time sensing data
Yan et al. (2025b)	City-level DT requirements from stakeholder perspectives	Stakeholder-based data architecture	Survey/interview data; urban infrastructure datasets
Fire Emergency Response			
Bi et al. (2018)	Digital fire platform using BIM and GIS integration	Data layer; component layer; application layer; presentation layer	Vector data; DEM data; fire resources data; 3D model data; fire service data
Kim et al. (2019)	Wildfire digital twin framework using interactive wildfire spread simulator	IoT server; wildfire-digital twin (WT-DT) service platform (IoT-DT interworking function; WT-DT lifecycle management; interactive wildfire (I-WF) simulation engine; I-WF simulation interface); WF-DT instance pools (WF-DT instance, wildfire visualization, WF-DT management)	IoT Sensing data; GIS data; landscape data (elevation, canopy layer, fuel model, slope)
Isikdag et al. (2008)	Site selection and fire response management GIS	Foundation layer; physical architecture layer; human–computer interaction layer; data management layer; problem domain layer	Environmental data; geospatial data; BIM data compatible with CAD and GIS data; block-level GIS data
Huang et al. (2024)	Wildfire DT for detection, simulation, and prediction	Sensor fusion; machine learning; layered architecture	Satellite, UAV, and ground-based sensors
Li et al. (2024)	Review of DT for wildfires	Systematic evaluation of components (sensors, models)	Literature-based data classification
Urban Emergency management			
Tashakkori et al. (2015)	3D indoor/outdoor spatial model for indoor emergency response facilitation	Emergency simulation model input layer; Platform layer; 3D system function layer (visualization, navigation, spatial analysis, situational awareness)	Building; storey; physical and theoretical bounded areas; environment detectors; emergency utilities; fire utilities; material information; and road network data
Lochhead and Hedley (2019)	Virtual evacuation simulations in real-world built environments	Mixed reality application layer; geographic application layer; emergency management application layer; AR for the emergency management layer	Smartphone GPS data; user behavioural data; real-time virtual data; evacuation data
Yu and He (2022)	DT-driven intelligence for disaster prevention and mitigation for infrastructure	Data layer; object layer; technology layer; connection layer; service layer	CAD drawings; BIM models; preliminary simulation models; maintenance and disaster data (e.g., collected by sensors, UAVs, laser scanners, satellites, robots); catastrophe data (e.g., collected by corresponding simulation models)
Gong et al. (2025)	Parallel simulation in urban underground DT	Parallel computing; prediction algorithms	Simulation data from underground utilities

Unlike individual entity-level DT, the DT framework at the city level represents a vast, interconnected system comprising various entities, infrastructures, and human dynamics. This complexity demands a multifaceted, layered, and spatially aware approach, where GIS emerges as a central pillar. GIS provides the spatial foundation, allowing for the accurate representation and interrelation of various urban elements. Meanwhile, dispersed across the city, IoT devices offer continuous data streams and real-time dynamism. The convergence of GIS and IoT creates a dynamic, multi-dimensional DT of the city, offering insights not just into individual entities but also their interplay, dependencies, and the cascading effects of events or decisions. In the context of smart resilient cities, this DT framework assists in identifying vulnerabilities, simulating potential urban challenges, and enabling proactive, data-driven urban planning and response.

**Fig. 1:**
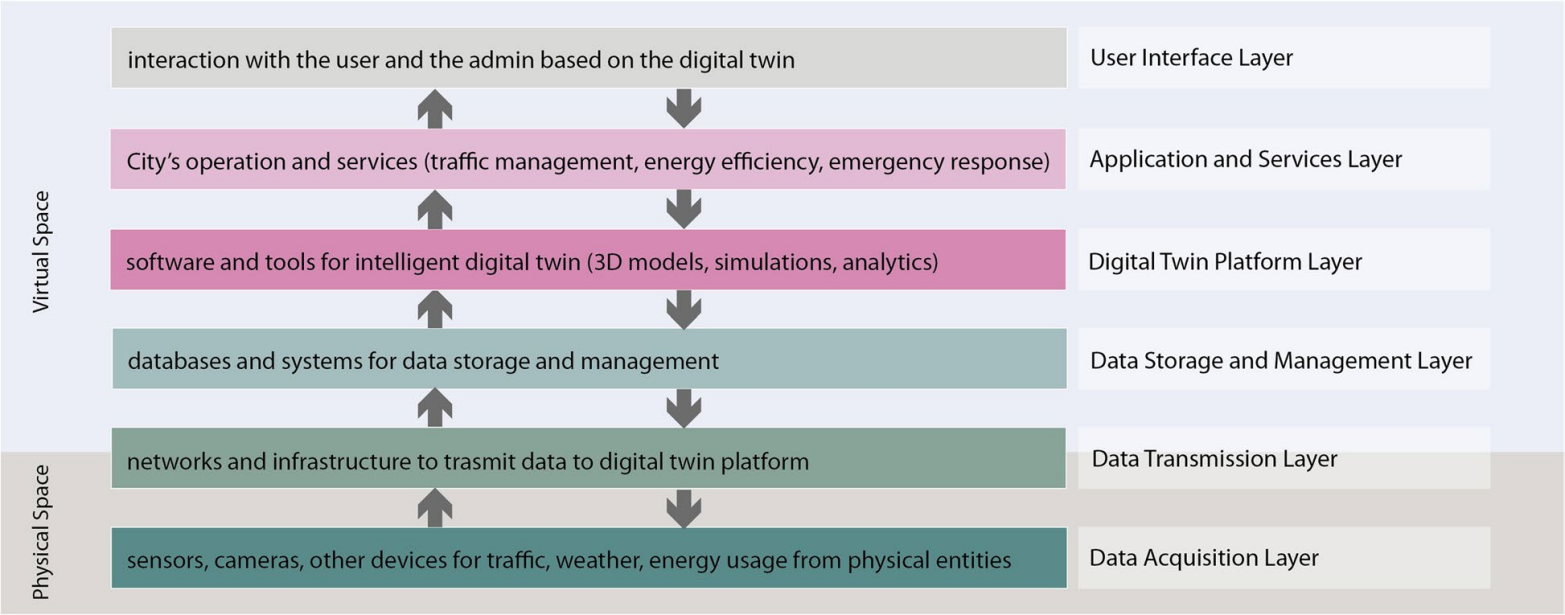
Hierarchical architecture of digital twin framework in smart cities

A smart city DT framework typically consists of components related to sensors and devices for collecting data, a network for transmitting and analyzing data, a DT platform for creating and maintaining virtual models of the city, and applications for using the data and models to improve the city's operations and services for the stakeholders. The architecture can vary depending on the specific implementation, but it generally includes the following layers (Fig. 1):

- Data acquisition: This layer includes sensors, cameras, and other devices that collect data about various aspects of the city, such as traffic, weather, and energy usage.
- Data transmission: This layer includes the networks and infrastructure to transmit data from the acquisition devices to the digital twin platform.
- Data storage and management: This layer includes the databases and systems that store and manage the data collected from the acquisition devices.
- Digital twin platform: This layer includes the software and tools used to create and maintain the digital twin of the city, including 3D models, simulations, and analytics.
- Applications and services: This layer includes the various applications and services that use the data and models from the digital twin to improve the city's operations and services, such as traffic management, energy efficiency, and emergency response.
- User interface: This is the layer where the user or the admin can view and interact with the data and models provided by the digital twin.

### A framework for DT-driven fire and smoke management

**Fig. 2:**
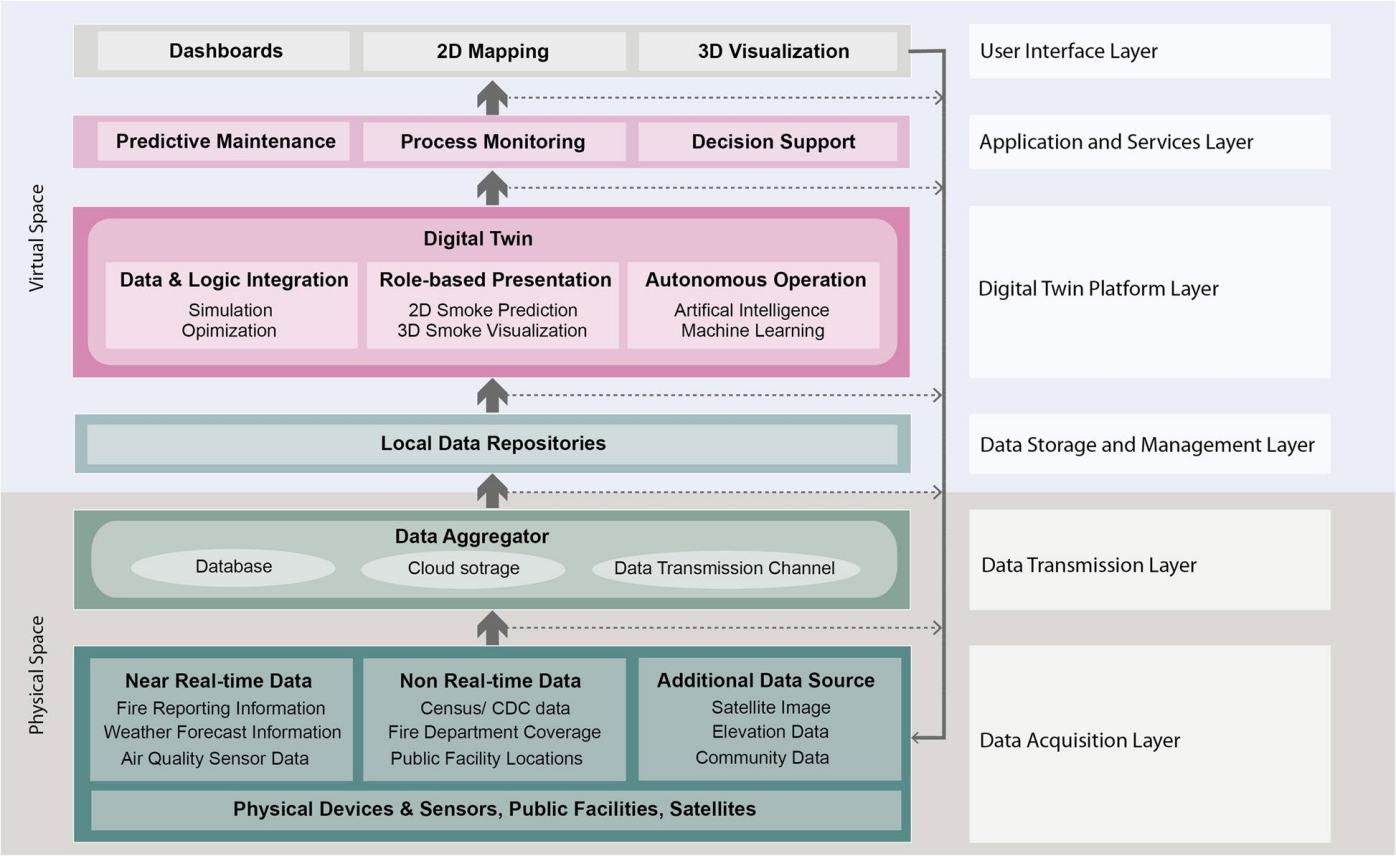
Multi-layered development framework of Digital Twin (DT) for comprehensive fire and smoke management in urban settings

As a conceptual framework for the DT, the initial setup was to review the practical applicability of the outcome and then the information utilization and technology integration required for fire and smoke management systems. The intended stakeholders included the local government and communities, and the expressed need was for a technically simple and practically efficient framework for fast assessments and public communication. Figure 2 briefly outlines the development framework of DT for fire and smoke management as an architecture of six layers.

- Data acquisition: The data relating to community fire and smoke management are divided into three categories: near-real-time data, non-real-time data, and additional community information. The near-real-time data includes fire reporting information, weather state and forecast information, and air quality data collected by physical IoT sensors (presently, and satellite, in-situ data model fusion in the near future). Non-real-time data include population information from the Census Bureau, the Centers for Disease Control and Prevention (CDC)’s chronic disease data, and points of interest (POI) regarding public facility locations. Additionally, satellite images, elevation data (from the digital surface model (DSM)), open street map road network, landcover layer, building footprints, and community infrastructure data are collected at this level.
- Data transmission: The data collected from physical devices, sensors, and public facilities are transmitted through the data transmission channel. It is a medium for sharing information between the data acquisition layer to the data storage and management layer in local data repositories. Fast transmission speed, robust compatibility, steady transmission, and cheap cost are required in a data transmission layer for DT systems (Yu & He, 2022; X. Zheng et al., 2022). On top of that, we adapted diverse data transmission channels into the Fire and Smoke DT system by considering the suggested requirements. Transmission technologies used in this study include Bluetooth, Wi-Fi, and satellite. The fire and burn information are secured by the Austin Fire Department and are transmitted to the team through a website.
- Data storage and management: Considering the data heterogeneity, the data transmitted from the physical spaces are integrated and stored in local data repositories. This is from the perspective of data privacy, security, and preparing the data for the DT pipeline. To provide the information for desired services through DT, diverse data storage and sharing solutions, including physical data servers, private cloud storage, and decentralized distributed file systems, were utilized for large volumes of data.
- Digital twin platform: Data fusion, prediction, and autonomous operation technology are three centerpieces of the DT platform layer. First, data and logic integration are utilized for fire and smoke simulation and optimization for decision-making at various stages of the process by transforming real-world needs into computer language and driving the solution synthesis in the desired direction. Second, a 2D smoke prediction model is developed and integrated into the mapping tool. Combining weather forecast data, building elevation data, and 3D geometry, we utilized Blender, the open-source 3D graphic software tool, to conduct 3D smoke visualization. For developing the 2D and 3D smoke prediction models, we used machine learning technologies to operate the near-real-time emulation.
- Applications and services: The primary purpose of this layer is to transform the DT layer results into services that meet the needs of target users (stakeholders). The service requirements in fire and smoke management include predictive maintenance for the city infrastructure, fire incident and smoke falloff process monitoring for affected communities, decision support for physical entities, as well as broader community education.
- User interface: The user interface layer facilitates communication between services the DT platform provides through end-user interfaces. Through visualized 2D mapping, web-based, interactive dashboards for fire practitioners and citizens are created to check fire and smoke spread in near-real time. The map is also converted to 3D city structures using OpenStreetMap, showing the 3D simulated smoke outputs to the end-users. The outputs are reviewed by the experts from Austin’s fire department before being released to the community.

### Data fusion, preprocessing, and performance

**Fig. 3:**
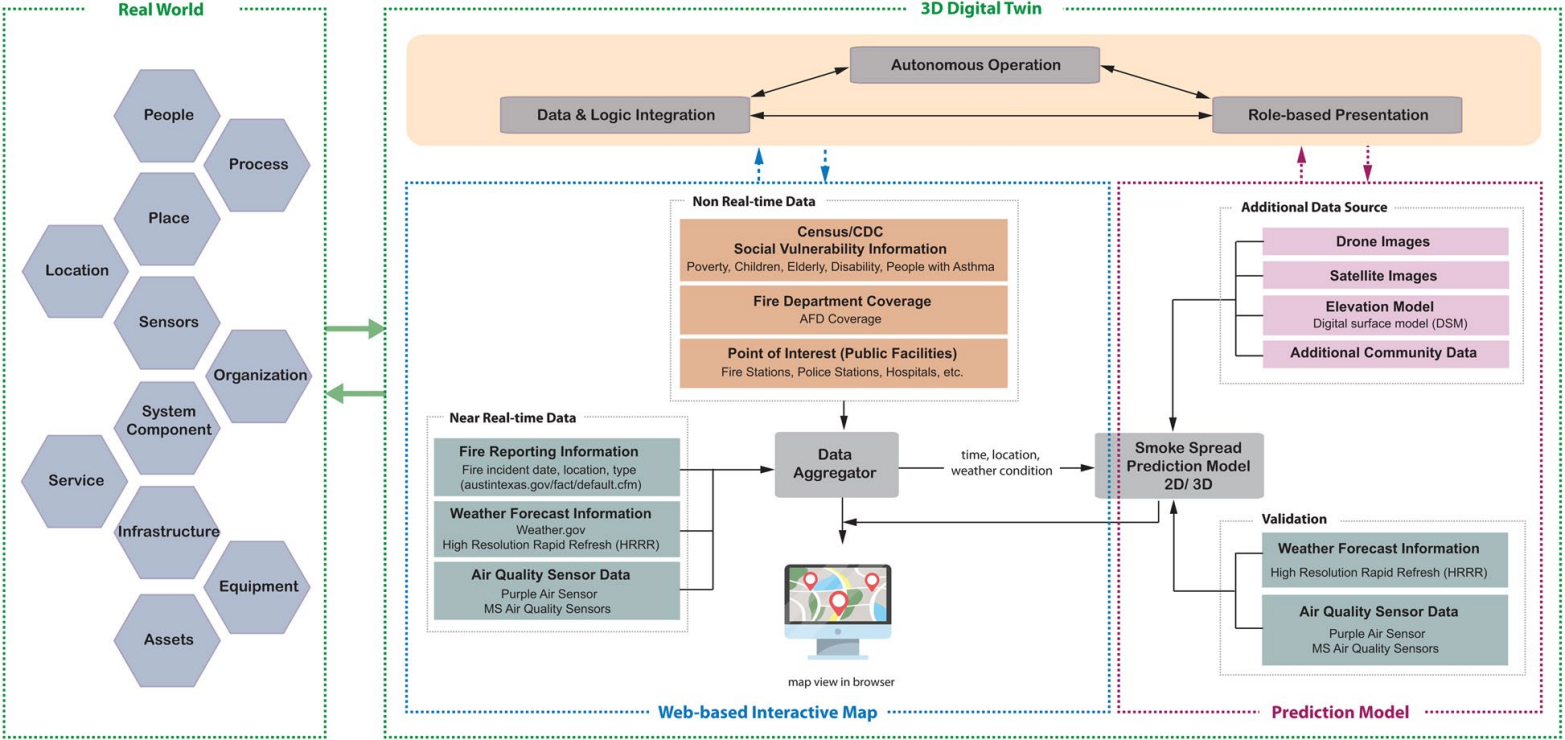
Data-driven framework and interactive service scenario for fire and smoke management using 3D digital twin technology

A core challenge in a city-scale DT is the integration of heterogeneous data. FireCom’s data aggregator (Fig. 3) is designed to resolve mismatches in temporal and spatial resolutions and to ensure data quality through a rigorous preprocessing workflow.

#### Data preprocessing and fusion

To handle data from over 20 different agencies and municipalities, our backend system employs a multi-stage workflow:

1. Schema Harmonization: Fire incident data from varied sources (e.g., HTML tables, RSS feeds, plain text, JSON) are automatically parsed and mapped into a unified schema. This standardized format captures key details: incident name, date, longitude and latitude coordinates, street address, and reporting department.
2. Temporal Synchronization: All timestamps are converted to Coordinated Universal Time (UTC) and aligned to an hourly update frequency. This ensures that near-real-time data from fire departments, weather services, and air quality sensors are temporally consistent for modeling.
3. Spatial Integration and Validation: Incident coordinates undergo validation to filter out errors. Addresses are geocoded using the Google Geocoding API when coordinates are missing. The validated locations are then intersected with census tract boundaries to integrate demographic and vulnerability data.

#### Multi-source fusion for smoke and risk estimation

FireCom integrates heterogeneous real-time and contextual data sources through a rule-based fusion framework designed to overcome disparities in spatial granularity and temporal frequency. The system ingests fire incident reports from emergency dispatch systems with point-level spatial resolution and a 3-min update frequency, providing the initial anchor for potential smoke events. These incident locations are then cross-referenced with localized PM2.5 concentrations from the PurpleAir sensor network, which offers hyperlocal coverage (100–500 m) and refreshes every 80–120 s. To estimate potential smoke dispersion, FireCom incorporates hourly wind forecasts from the NOAA High-Resolution Rapid Refresh (HRRR) model, which provides 3 km spatial resolution and hourly updates. All real-time data streams are harmonized onto a common hourly temporal grid. PM2.5 values are resampled using rolling averages, fire incident data are aggregated or retained as centroids within 60-min windows, and HRRR wind fields are interpolated using ordinary kriging, which effectively models spatial autocorrelation and provides statistically optimal estimates in heterogeneous urban environments. This approach ensures alignment with the finer spatial scale of sensor and incident locations.

#### System performance and latency

For a DT to be effective in emergency response, its performance and latency are critical. The FireCom platform was tested over a six-month period, processing over 10,000 fire incidents. The system achieves the following performance metrics in Table 2:

**Table 2:** FireCom system performance metrics for real-time smoke modeling

Metric	Specification/Value
System-wide Update Interval	Hourly (60-min intervals)
Data Source Update Frequency	• Fire Incidents: every 3 min • PM2.5 Sensors: every 80–120 s • HRRR Wind Fields: hourly updates
2D VSmoke Processing Time	< 10 s per fire incident
3D MantaFlow Simulation Time	20 s—1 min per incident (offline)
End-to-End Latency (2D Pipeline)	2–5 min from fire report to visualization • Ingestion (< 30 s) • Schema mapping & validation (~ 1 min) • VSmoke modeling (< 10 s) • Visualization/rendering (< 2 min)

The 2D pipeline produces visualizations within 2–5 min of a reported fire, whereas the 3D pipeline, which requires 5–15 min per simulation run depending on urban complexity, is updated on an hourly cycle to provide detailed analysis. The current deployment did not capture full quantitative metrics for data completeness or system availability. Preliminary observations during the six-month pilot indicate that ingestion failures and downtime were rare, typically caused by network issues, malformed external data, scheduled maintenance, or upstream API outages. Future work will implement continuous monitoring to establish precise performance benchmarks.

## Service scenario for fire and smoke management

The DT framework for fire and smoke management is tested by modelling a test case in the City of Austin. The DT application and its platform were established to provide targeted and timely information about smoke occurrences to fire practitioners and local communities to improve health and life safety (Lewis et al. 2024).

This real-time fire and smoke occurrence tracking platform is named ‘FireCom’ and is intended to serve as a foundational framework for community awareness and an educational dashboard. The FireCom’s technical architecture builds off the monitoring and impact prediction system. Figure 3 shows the data processing models and service scenarios for fire and smoke management. Real-world information interacts with a 3D DT based on a data aggregator and a smoke spread prediction model. All forms of data acquired from the real world are stored and controlled in a data aggregator to be shown in a browser as a web-based interactive map. The input data is then utilized to estimate smoke spread. On top of that, 3D DT is retained through autonomous operation, data and logic integration, and role-based presentation.

As illustrated in Fig. 3, we delve into the triad of foundational elements that comprise our framework: the Web-based Interactive Map for Fire Incidents, the Smoke Spread Prediction Model, and the 3D Digital Twin for Fire and Smoke Management. The first component provides a real-time, dynamic visualization of active fire incidents, enhancing situational awareness and empowering emergency responders with prompt, informed decision-making capabilities. Second, our predictive model leverages cutting-edge algorithms combined with meteorological data to forecast the potential trajectory and concentration of smoke, playing a pivotal role in proactive evacuation planning and ensuring the safety of susceptible populations. Finally, our 3D DT offers insight into the intricate dynamics of fire and smoke propagation amidst urban structures by emulating real-world urban environments in a digital space. It autonomously integrates diverse data sources and computational logic, ensuring timely insights tailored to individual users, thereby maximizing its relevance and functionality across various stakeholders.

### Web-based interactive map for fire incidents

The DT relies primarily on the geographical setting and the information collected in near-real-time. We collected the real-time information needed for the simulation, including real-time fire reports transmitted from the Austin Fire Department (Austin Fire Department, n.d.). Regarding weather, local conditions and forecast information, wind and other meteorological data from the National Weather Service (National Weather Service, n.d.) were collected for each fire’s reported location. We gathered real-time sensor data from all surrounding air quality sensors using PurpleAir (PurpleAir, n.d.) and Microsoft Air Quality Sensors (MSN, n.d.). That information was formatted for a live presentation on an interactive map. Thus, the end-users can view fire location (marked as an orange flame if active burn or a burnt grey flame if inactive), wind speed, wind direction, temperature, and weather forecast, and air quality information (real-time PM2.5 Air Quality Index and up to one-week average) and pan the map for data from the previous three days or to a specific date to examine additional information.

**Fig. 4:**
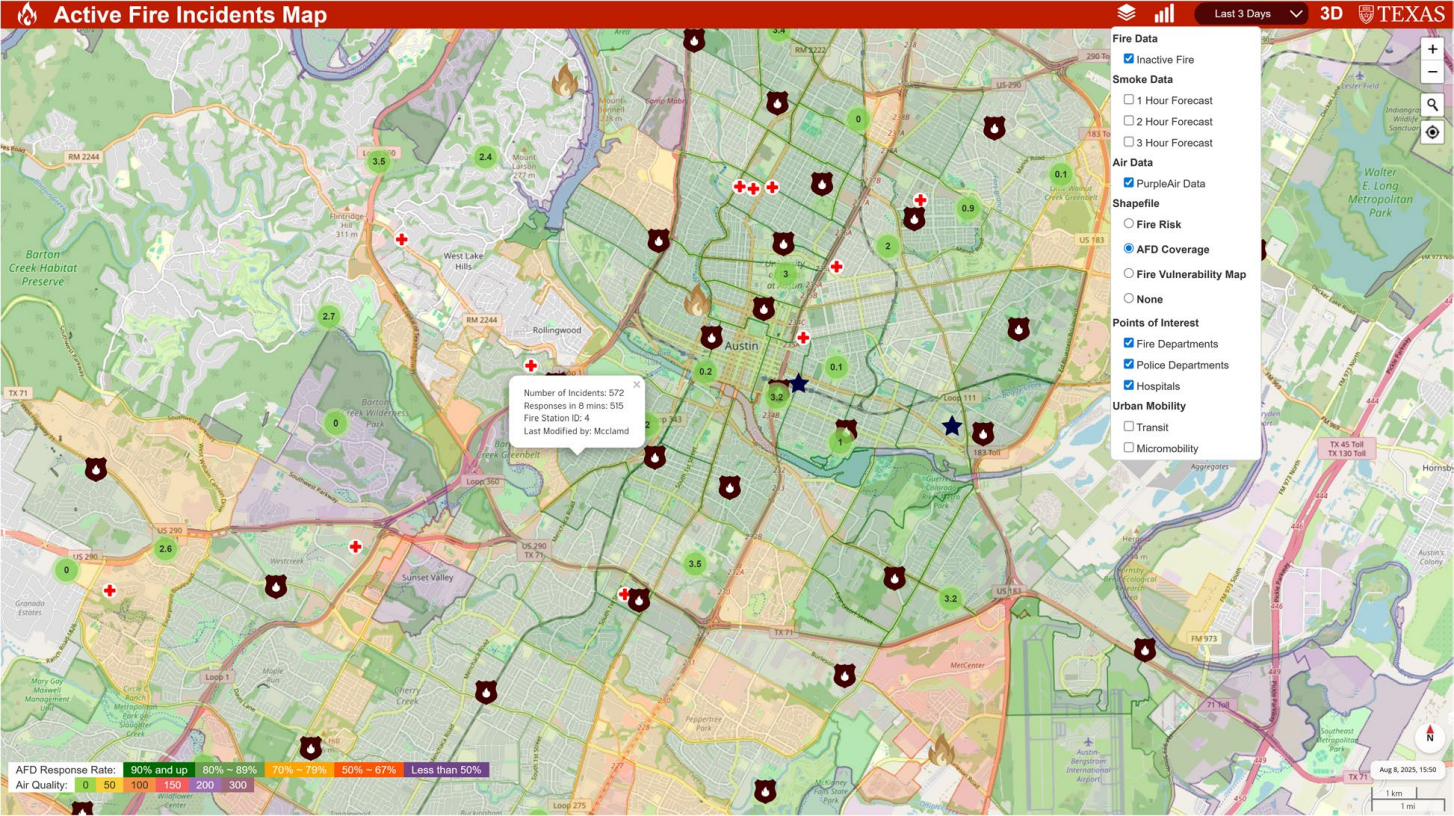
A snapshot of the 2D platform of FireCom in Austin, Texas. Burnt grey flames indicate inactive fire incidents. A white pop-up box displays the number of incidents within the fire department’s coverage area, response times, and allocated fire stations. Dark brown, blue star, and red cross icons symbolize fire department, police department, and hospitals, respectively. Each type of public facility can be displayed by selecting the corresponding toggle in the drop-down menu at the top right. Census tracts are color-coded by fire response rate: dark green indicates higher response rates, while purple represents lower response rates

Other essential data used in this research were non-real-time fields: census data (U.S. Census Bureau, n.d.) and the CDC data (Cdc, 2021) that includes the census-tract level fire vulnerability information (i.e., families below the poverty level, children, elderly, people with disability or respiratory diseases, and other information of interest in the future). Also, the total number of fire incidents over seven months was aggregated by census tract to assess fire risk potential, with fire department coverage evaluated using an eight-minute response time threshold to ensure efficient emergency response, aligning with the spatial analysis of firefighting pressure distribution in urban settings to optimize service equity (J. Li & Hu, 2025). Finally, the public facilities, including the locations of fire departments, police departments, and hospitals, were included in the interactive map. A snapshot of the 2D platform is shown in Fig. 4.

The technical steps for establishing the 2D platform of FireCom are given below:

- Real-time fire incident data was procured from Austin’s active fire incident page (Austin Fire Department, n.d.).
- A unified schema was established to capture key details: incident name, date, longitude and latitude coordinates, street address, and reporting department.
- Scripts were crafted to seamlessly convert diverse formats such as HTML tables, RSS feeds, plain text files, and JSON documents into the devised unified schema.
- By juxtaposing the latest acquired data with the information from previous retrievals, fire statuses were persistently updated.
- Fire incident data underwent intersection processes with a tract-level map of Austin to facilitate the determination of fire risk in areas.
- We queried weather data (i.e., temperature, weather forecast, wind direction and wind speed) and extracted information from the National Weather Service (National Weather Service, n.d.) through an API.
- An assemblage of time-independent data was curated, incorporating elevation maps, building footprints, and points of interest (i.e., fire departments, police departments, hospitals) collected from the Austin Open Data Portal (City of Austin, n.d.).

### Smoke spread prediction model

Based on the time and location of fire incidents and weather conditions collected in the data aggregator, we developed a 2D smoke spread prediction model that can be overlapped with a 2D map. Based on the time and location of fire incidents and weather conditions collected in the data aggregator, we developed a 2D smoke spread prediction model that can be overlapped with a 2D map. Predicting the spread of smoke is a complex yet essential endeavor, combining real-time fire data, meteorological insights, and advanced computational modeling. Firstly, all fires reported to the fire department were tabulated and plotted per location on a map using fire burn coordinates. Concurrently, for each reported coordinate, we source real-time meteorological conditions from the National Weather Service (National Weather Service, n.d.). This includes parameters such as wind direction, wind speed, and temperature. Augmenting this, predictions derived from the High-Resolution Rapid Refresh (HRRR) model (NOAA Research, n.d.) obtained from the National Oceanic and Atmospheric Administration (NOAA) are seamlessly integrated, offering a nuanced understanding of potential meteorological shifts.

The next phase focuses on smoke dispersion modeling. Leveraging the integrated dataset, the "VSmoke" model (FCAMMS,  n.d.), developed by the Georgia Forestry Commission for predicting smoke from controlled fires (Lavdas, 1996a, 1996b), is employed to gauge smoke dispersion. VSmoke offers outputs that depict peak hourly particulate matter (PM2.5) concentration grounded in the Gaussian plume model framework (Arya, 1999). While VSmoke was originally developed for controlled forest burns, it is utilized in FireCom as a rapid, first-order approximation model suitable for real-time alerts. Its Gaussian plume assumptions, while a simplification in complex urban terrains, provide a computationally efficient method for generating initial smoke trajectory forecasts. We acknowledge its limitations in capturing urban canyon effects, which motivated our development of a more sophisticated 3D simulation for detailed analysis. Based on Laydas 1996a, the concentration equation for a singular pollutant source, as dictated by the Gaussian plume model within the specified constraints, can be articulated as follows:

$$C= \frac{Q}{\pi {\sigma }_{r}{\sigma }_{z}U}exp\left[-\frac{1}{2}{\left(\frac{H}{{\sigma }_{z}}\right)}^{2}\right]$$

Where

C = concentration due to the source in micrograms per cubic meter,

Q = source strength emission rate in micrograms per second, 

${\sigma }_{r}$ = horizontal dispersion coefficient in meters (a function of atmospheric stability and downwind distance),

${\sigma }_{z}$ = vertical dispersion coefficient in meters (a function of atmospheric stability and downwind distance),

U = transport wind speed in meters per second (as input), and

H = plume height in meters (a function of atmospheric stability and downwind distance).

**Fig. 5:**
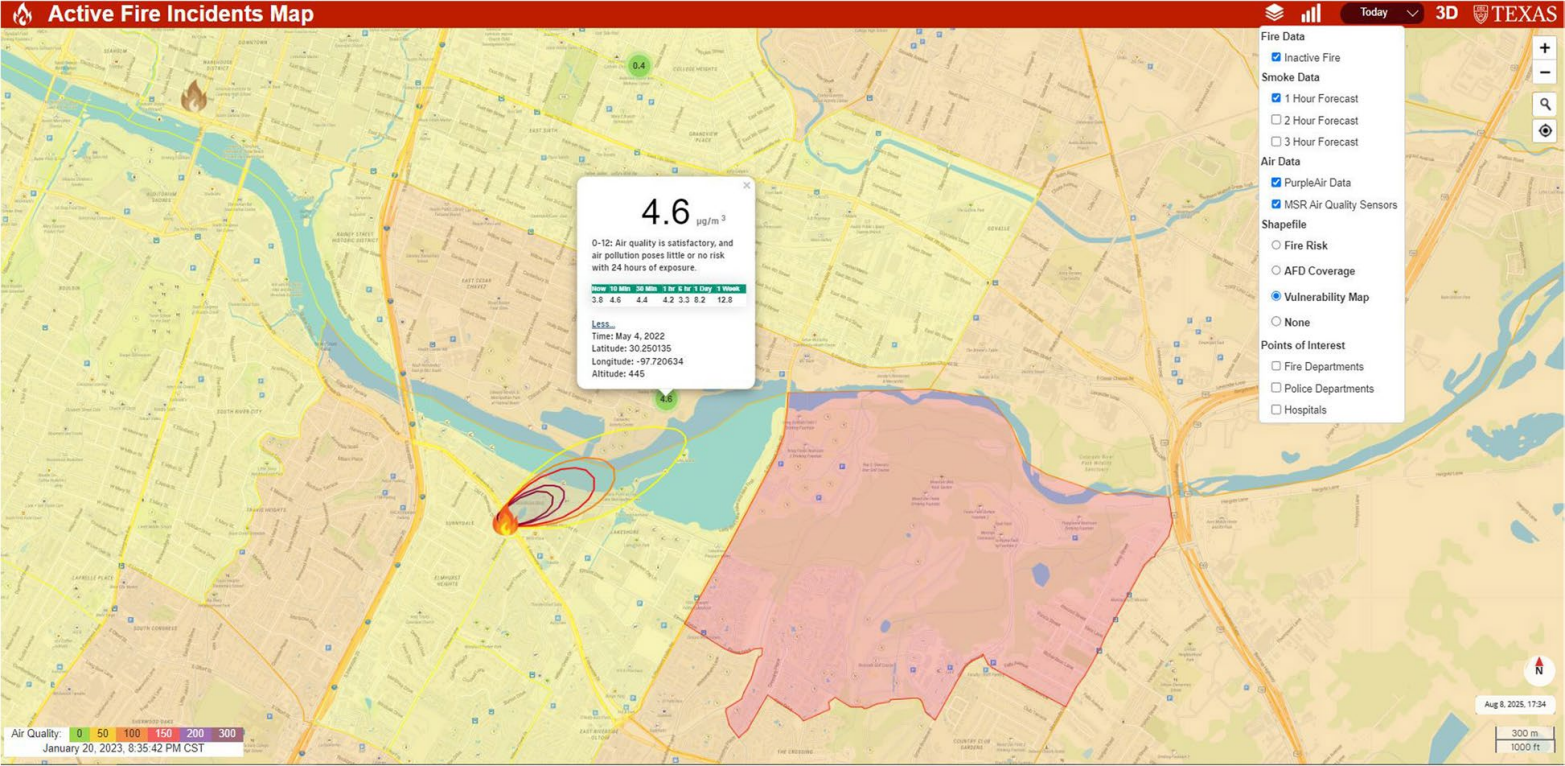
A snapshot of the smoke spread prediction outcome with a burn reported on 1723 E Riverside Dr., Austin, Texas. The yellow, orange, red, purple, and maroon circles from fire symbolize different levels of concern: moderate, unhealthy for sensitive groups, unhealthy, very unhealthy, and hazardous, respectively. It predicts ranges of up to three hours and may be seen by choosing an hour prediction from the drop-down menu on the top right. “4.6” in the white box represents the real-time PM2.5 Air Quality Index

To apply this model, Pasquill atmospheric stability classes (ranging from A—very unstable to F—moderately stable) are determined from meteorological data (wind speed, solar radiation). The horizontal and vertical dispersion coefficients (${\sigma }_{r}$ and ${\sigma }_{z}$) are functions of these stability classes and downwind distance, calibrated based on the Pasquill–Gifford empirical curves (Briggs, 1973; Turner, 2020), which are widely used for open terrain. The combination of high-resolution datasets, especially those from the HRRR, with the VSmoke model ensures predictions for up to three hours. The visualization is facilitated through contour values, and the associated color schemes are mapped in line with PM2.5 thresholds set by the Air Quality Index (AQI). Figure 5 shows the smoke dispersion concentration for a burn reported on Riverside Dr, Austin, Texas.

The technical steps for developing a smoke spread prediction model are given below:

- A Gaussian smoke prediction model, grounded in the VSmoke particulate matter (PM2.5) concentration originating from the Georgia Forestry Commission (GFC), was utilized.
- Real-time weather parameters (i.e., wind speed, wind direction, and humidity) and locations of the fires (i.e., latitude and longitude data presented in decimal degrees) served as inputs for the VSmoke model.
- Consideration was given to distinct fire types and their intrinsic characteristics.
- In the VSmoke model, contour values, along with their corresponding colors, align with the PM2.5 thresholds defined by the Air Quality Index (AQI), referenced by the AirNow platform (AirNow, n.d.).

### 3D digital twin for fire and smoke management

As VSmoke does not explicitly consider the geometry of nearby structures, it can be observed that the predicted smoke floats over the structures in the immediate vicinity of its path. A review of the conditions that might cause such flow was deemed unrealistic. To address this, we developed a 3D representation of the city and the smoke (Kamath et al., 2022) using Blender with OpenStreetMap (OSM) 3D building footprints and height data (Over et al., 2010). This approach constructs approximate models of each structure, enabling building-resolved flow simulations.

 In our 3D visualization, we adopted Blender, which enables interaction with Python and provides access to advanced fluid simulation tools like MantaFlow. While offline rendering engines like Blender are not designed for the instantaneous feedback of gaming engines (e.g., Unity or Unreal), this choice was deliberate. It prioritizes simulation accuracy and physical realism over rendering speed, which is more critical for decision support in emergency management in emergency management.

To accurately model smoke dispersion within the complex urban morphology, a sophisticated simulation is required. Our algorithm uses an automated Python script to create a fluid simulation at the precise longitude and latitude of a fire event. It employs a Large Eddy Simulation (LES)-like approach to handle urban wind turbulence, where large-scale eddies are directly resolved on a grid of 128 × 128x128 voxels, with adaptive refinement increasing the effective resolution up to 256^3^ near building surfaces. These building models are configured as solid boundaries with no-slip walls, forcing the simulated smoke to realistically interact with urban geometry. Simulations for a 1 km^2^ domain are executed on an NVIDIA RTX 4090 GPU (24 GB VRAM), with typical run times of 5–15 min per fire event, making them suitable for hourly 3D updates rather than instantaneous, interactive rendering.

In terms of precision, the Blender 3D city model incorporates not only detailed building geometries but also a topographic height map from OpenTopography (accurate to 30 m) (OpenTopography, n.d.) as the DT base layer for smoke simulation. The simulation results are visualized in-browser via MapBox GL, which renders the same OpenStreetMap 3D tiles alongside the MantaFlow smoke plumes in a vector-tiled format.

While this 3D extension significantly enhances realism for urban environments, certain modeling uncertainties persist. The underlying VSmoke component maintains Gaussian plume assumptions optimized for open terrain, and although MantaFlow simulations are building-resolved, they operate under idealized boundary conditions with limited meteorological forcing. High-fidelity urban CFD models, (i.e., OpenFOAM, PALM-4U, and QUIC-URB) could provide more accurate turbulence resolution but demand computational resources incompatible with near-real-time emergency response requirements. Our approach achieves an optimal balance between physical fidelity and operational efficiency, enabling FireCom to enhance 2D outputs with building-aware dispersion predictions that directly support targeted emergency response strategies.

In brief, this explicit treatment of building geometry and street canyon channelling enables FireCom to capture dispersion behaviors unique to dense urban environments. Such urban-specific aerodynamic effects are typically absent from wildfire-oriented DT frameworks, which are optimized for open terrain conditions.

**Fig. 6:**
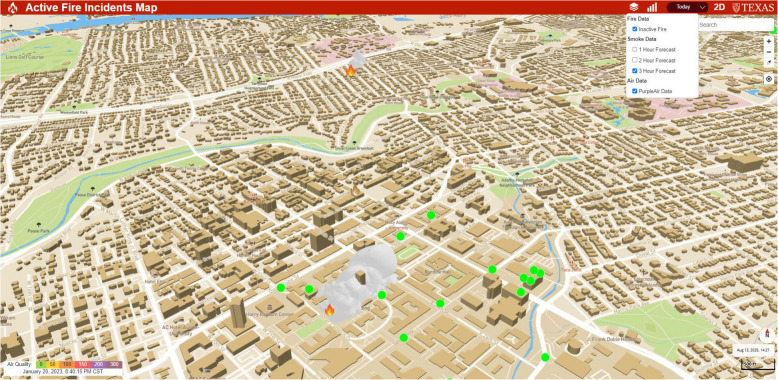
A snapshot of the 3D visualization of the city topology, building structures, and smoke simulation. The orange flame shows the approximate location of the localized fire or burn, and the grey plume around the fire is representative of the smoke spread. The map shows the area centered around the university campus

Figure 6 shows a snapshot of the 3D visualization of the city topology, building structures, and smoke simulation. In Fig. 6, we can see two burn sites: one in the top center (a brush fire across Shoal Creek) and the other in the bottom center (the tower fire at the university campus) of the map. This example is for visualization purposes, illustrating how smoke disperses and interacts with urban morphology, and does not depict an actual fire event. Grey plumes were generated through Mantaflow in Blender and overlayed with 3D city topology and building data. The map's green, blue, and pink areas represent green space, waterbody, and hospital areas, respectively.

**Fig. 7:**
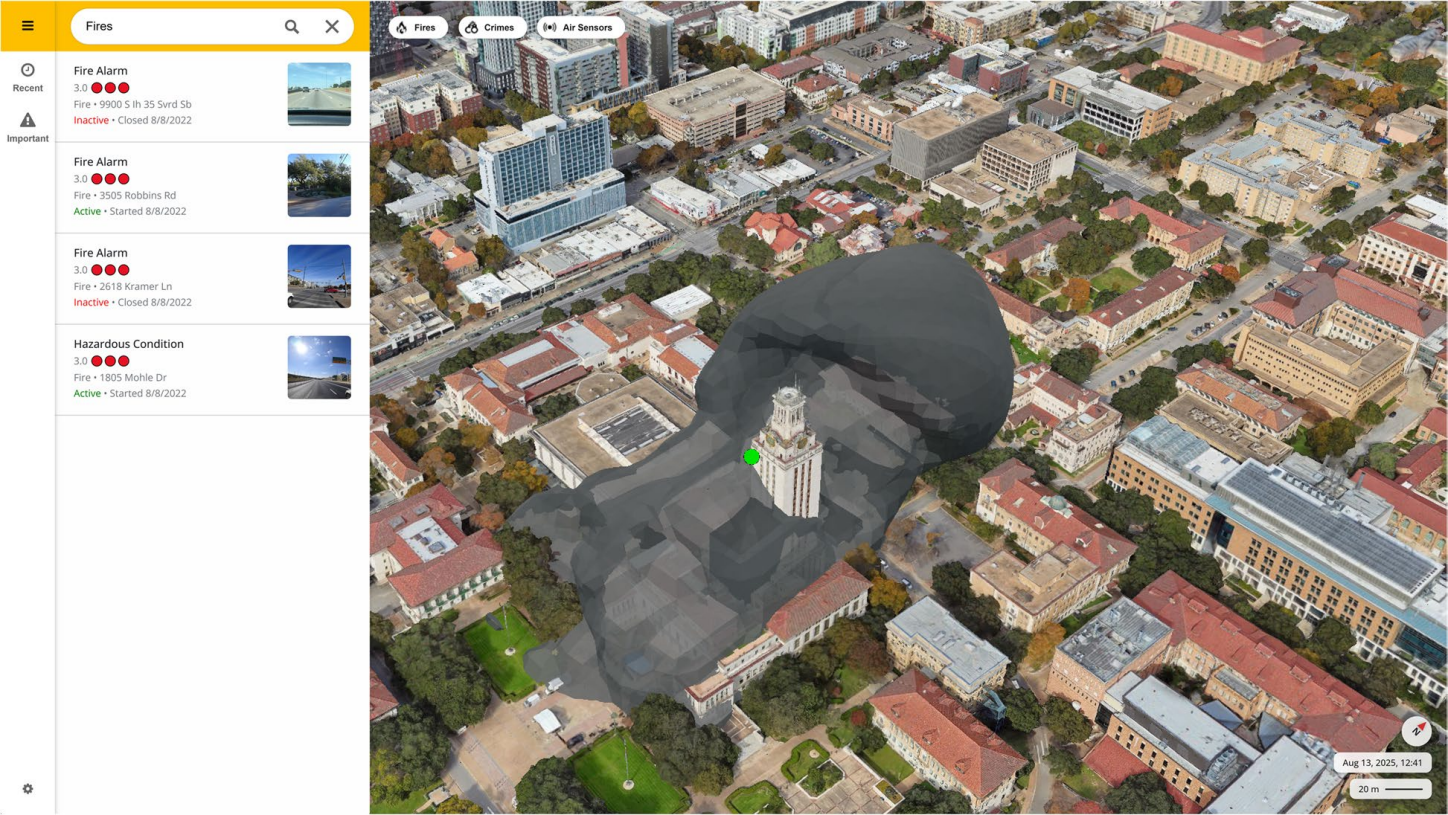
An advanced 3D visualization of fire and smoke simulation using textured building models and enhanced urban context, incorporating realistic building geometries and surface textures and offering a more operationally relevant representation of the cityscape. The simulated smoke plume is shown dispersing around the university campus, illustrating detailed urban morphology for accurate smoke forecasting

The technical steps for establishing a 3D digital twin for fire and smoke management are given below:

- Leveraging both Blender and OpenStreetMaps (OSM) 3D, a 3D-rendered building footprints was established to overcome the inherent limitation of 2D models.
- The model was further enhanced by integrating it with a precise topographic height map of Austin, sourced from OpenTopography.
- A BlenderPy script (Gubala, n.d.) was crafted to facilitate the creation of a smoke fluid simulation, ensuring alignment with specific coordinates within the 3D city model, using parameters comparable to those in VSmoke.
- MantaFlow, a state-of-the-art fluid simulation framework, was leveraged to achieve more realistic and dynamic smoke dispersion simulations in the 3D city model.
- An API was formulated to initiate fluid simulations of fires in Blender when demanded.
- The program's outputs were exported and reported hourly marks as Filmbox (.FBX) object files.
- Visual representation with MantaFlow simulations was found on a vector-tiled 3D map courtesy of MapBox GL (Fig. 7).

### Model validation and evaluation

A critical component of developing any predictive model is empirical validation. Validating a smoke dispersion model in a dynamic urban environment, however, presents a significant challenge. The sporadic and unpredictable nature of urban fires combined with the sparse distribution of stationary air quality monitors makes it highly improbable that a sensor will be opportunistically located in the direct path of a smoke plume from a given incident. Our analysis of sensor networks in Austin and other major cities confirmed this "needle in a haystack" problem; even in cities with thousands of sensors, documented fire events rarely occurred close enough to a monitor to provide useful validation data.

To overcome this limitation, we conducted a controlled field experiment in collaboration with the Austin Fire Department. By leveraging a scheduled prescribed burn for firefighter training, we were able to strategically deploy a network of mobile air quality sensors at varying distances downwind of a known fire source. This allowed us to collect ground-truth PM2.5 data and directly compare it to the forecasts generated by FireCom’s 2D smoke prediction model.

The experiment validated the model's ability to accurately predict the spatial extent of different Air Quality Index (AQI) categories. The model achieved an overall categorical match rate of 72.7% when comparing predicted AQI zones to ground-based PM2.5 measurements, with most mismatches falling into an adjacent AQI category. The typical spatial deviation between predicted and observed category boundaries was minimal, no more than 80 feet, demonstrating strong spatial fidelity for emergency communication purposes. While this categorical agreement and low spatial offset provide a robust preliminary validation in the absence of formal error statistics, they serve as functional proxies for conventional performance metrics such as RMSE or MAE in this early-stage deployment. Future work will extend this validation using historical Austin fire events to compute quantitative indicators across multiple scenarios, thereby strengthening the statistical rigor of model evaluation.

Furthermore, this controlled experiment highlighted the importance of the 3D model. Several sensors placed downwind but behind large buildings or other obstructive geometry registered no significant change in air quality, an outcome that the 2D model could not explain but was consistent with the smoke-blocking behavior predicted by the 3D MantaFlow simulations. This demonstrates the necessity of a dual-model approach: a rapid 2D model for immediate, widespread alerts and a detailed 3D model for analyzing the complex effects of urban morphology on smoke dispersion.

## Discussion

This paper investigates the DT framework presented in earlier literature, applies the DT architecture to fire and smoke management, and develops a 2D and 3D web-based fire and smoke monitoring platform.

Employing DT for urban resilience offers numerous benefits, notably the capability to simulate, analyze, and predict environmental risks, enabling proactive and informed decision-making. For urban planning and emergency management practitioners, the DT framework at the city level serves as a robust tool to visualize real-time scenarios, aiding in both short-term emergency responses and long-term urban development strategies. However, integrating DT into city frameworks involves challenges, including concerns regarding data accuracy, incorporation of multi-source datasets, scalability, and real-time responsiveness. Recognizing these challenges, we aim to address them in our future work by refining our DT model, ensuring its adaptability and scalability, and further collaborating with city stakeholders to ensure its applicability and relevance in real-world urban settings. Future work will also incorporate building materials, fire resistance ratings, and fire lane layouts into the 3D smoke dispersion model once suitable datasets become available, enhancing the current OpenStreetMap-based building geometries with material-specific attributes for more comprehensive urban fire risk assessment.

The novelty of the study includes three main advancements. First, all data used in this study, including fire incidents data, weather information, air quality sensor data, city data, census data, and neighborhood-level disease data, are open and establish a replicable methodology for any city with similar data. This generalizability means our approach can broaden DT's impact on fire and smoke management in various regions and scales. Second, this study effectively integrates and simplifies complex data fusion from different sources. Despite their complexity, we have incorporated data from multiple heterogeneous sources into processing and merged them coherently to retain a cohesive representation of the information. Third, "FireCom" offers 2D and 3D visualizations for enhanced communication. The 2D platform displays fire incidents, neighborhood risks, and public facility locations. In contrast, the 3D version gives advanced smoke predictions, overlaying topographical and structural details. Therefore, by presenting both 2D and 3D smoke prediction results, this study suggests that the use of smoke prediction depends on the specific requirements of the application and the desired level of accuracy, as well as the availability of data and computational resources.

Despite its novelty, both 2D and 3D components of FireCom involve modelling assumptions that influence uncertainty. The 2D VSmoke module relies on Gaussian plume formulations originally designed for open terrain, which may misrepresent dispersion in highly irregular urban flows. The 3D MantaFlow simulations explicitly incorporate building geometry and reproduce canyon-channelling effects but still operate with idealized boundary conditions and limited meteorological forcing. High-fidelity CFD frameworks could improve turbulence representation and micro-scale flow accuracy, though at the cost of computational performance critical for real-time emergency operations. Our hybrid strategy, using the 2D model for rapid citywide alerts and the 3D model for targeted, geometry-aware analysis, seeks to balance physical realism with operational speed.

In addition, validating the model for on-ground accuracy and reliability is one of the critical next steps in continuing this study. Specifically for the 3D smoke prediction model, one possible pathway for our ongoing and future work would be using air quality sensors to develop backward trajectories by measuring the reported air quality change and how far it was from each fire. The methodology for such approaches for large scale (10–100 km) scale is relatively well-developed (Mallia et al., 2020; Nguyen et al., 2022; Occhipinti et al., 2008; Shie & Chan, 2013). For this project, our next step is to utilize machine learning algorithms with data from PurpleAir monitors overlapping with smoke trails and validate our smoke prediction model by evaluating the air qualities caused by smoke. For this, the Austin Fire Department will invite academic researchers to participate in the controlled, prescribed burns. The researchers are installing more air quality sensors throughout the city so that the devices can capture local fires that are occurring nearby (Kaginalkar et al., 2022).

Our pipeline is the first phase in the development of DT, which will provide us with academic, technical, and practical field-based application potential for the future. Our data analysis platform is designed to assist the city in better understanding urban fire incidents and communicating with the public about the potential smoke spread via 2D mapping and 3D visualization. Residents may thus better understand if their physical environments are safe through fire and smoke detection, allowing municipal authorities and citizens to better prepare for fire emergencies. Preliminary stakeholder feedback gathered through multiple meetings with the Austin Fire Department (AFD) highlights the platform’s operational value. AFD noted the potential for improved public communication, awareness, and education, particularly for vulnerable populations such as individuals with asthma or allergies, through targeted air quality alerts and outreach to public schools and senior centers in areas with high asthma prevalence. They also emphasized the active map’s utility as a real-time warning system for smoke exposure and recommended maintaining a focused scope in the early deployment phase to ensure clarity and reliability. AFD further identified Austin Public Health (APH) as a key future partner to enhance the dashboard’s decision-support capabilities for public health interventions.

Building on this feedback, our smoke simulation models can further support intelligent decision-making. Historical fire incident cases can be used for smoke prediction and validation, enabling the fire department to respond more effectively, similar to the framework outlined for risk-informed dataset development in disaster and climate resilience (Fakhruddin et al., 2022). Finally, the integration of real-time data into a single aggregator is expected to enhance citizen safety and comfort. For example, the platform could be used to monitor air quality in communities with high rates of respiratory diseases and to identify potential associations between elevated urban fire risk and populations with COPD, asthma, or other illnesses. With smoke detection capabilities, the city could allocate EMS resources more rapidly through real-time monitoring. Moreover, web-based applications like FireCom facilitate efficient information exchange among multiple stakeholders (Kaginalkar et al., 2021), supporting both operational coordination and broader public engagement. It will make communication easier for the city, thanks to the web-visualized interface. Moreover, it will help build educational modules both within the classroom and for the general public, ultimately aiding the development of new knowledge as well as public services and policies that would not have been possible without real-time data integration, monitoring, and prediction of actual occurrences in physical environments. As the platform integrates near-real-time geospatial data such as fire incident reports and sensor readings, it is essential to consider data privacy and ethical implications. Although FireCom currently operates on publicly available datasets, future iterations will incorporate privacy-preserving measures, such as dynamic spatial aggregation (e.g., from point-level to block group), anonymization of sensitive locations (e.g., domestic violence shelters, emergency evacuation centers), and access control protocols for high-resolution data. These strategies will be developed in collaboration with city agencies to ensure that public-facing visualizations balance transparency with safety and privacy.

## Conclusion

The growing interest in fire digital twins reflects a broader trend toward predictive, AI-enabled disaster management. While recent efforts have achieved remarkable capabilities at regional (e.g., NASA’s Wildfire Digital Twin) and global (e.g., OroraTech) scales, our work addresses a crucial gap in applying such technologies at the city level, where real-time integration with urban systems and community-facing tools is critical. By incorporating 3D urban infrastructure data, live sensor feeds, and predictive smoke dispersion modeling, FireCom advances the state of digital twin design for urban fire and air quality risk. Its emphasis on accessibility, integration, and local operational use offers a replicable model for climate-resilient urban management.

This need is especially urgent in urban settings, where fires—both large-scale and localized—pose serious threats to public safety. Urban fires can cause tremendous physical, mental, and financial harm, requiring rapid and informed responses that depend on real-time situational data (Shuman et al., 2022). Beyond large-scale disasters, even small “nuisance” burns can degrade air quality and pose health risks to vulnerable populations (Mandalapu et al., 2024). These complex and layered challenges highlight the importance of city-scale tools like FireCom that not only track fire events but also anticipate their broader impacts. In this context, our study adapts digital twin smart city (DTSC) concepts for fire and smoke detection and management, with a focus on addressing the demands of effective, real-time communication for emergency response and community awareness. Accordingly, this paper explores the application of the DT framework towards urban resilience by developing an integrated platform for real-time fire and smoke tracking and prediction for communities. This platform is co-created through a partnership between academic researchers and field practitioners/the city fire department as stakeholders. The paper examines the pathway for establishing a fire and smoke 3D DT by deploying a near-real-time dataset and integrating non-real-time community information. The paper also aligns the efforts with previous DT models and discusses the challenges and opportunities for 3D DT in fire and smoke management.

Results include a comprehensive fire and smoke DT model for Austin, Texas, as well as the code and methods for replicating similar models for other cities worldwide. This study provides a reference case to adapt the theoretical concept of 3D DT smart cities and apply it to real-world fire and smoke management. We expect this study to help fire practitioners better protect and inform the citizens with targeted and timely information on fire incidents and ultimately help mitigate health and life safety impacts.

## Data Availability

The datasets generated and/or analyzed during the current study are available in the FireIncidentData repository, accessible at https://github.com/UrbanInfoLab/FireIncidentData. Additional data and resources can be found in our related GitHub repositories.

## References

[CR1] AirNow. (n.d.). *AirNow current air quality map*. Retrieved October 13, 2023, from https://gispub.epa.gov/airnow/?monitors=ozonepm&contours=none&panel=0

[CR2] Arya, S. P. (1999). *Air pollution meteorology and dispersion* (Vol. 310). Oxford University Press.

[CR3] Austin Fire Department. (n.d.). *Active fire incident page*. Retrieved October 13, 2023, from https://services.austintexas.gov/fact/default.cfm

[CR4] Bi, T., Wang, P., & Zhang, Q. (2018). *Design and implementation of digital fire control system based on BIM and 3DGIS*. 1810–1813. 10.2991/ifeesm-17.2018.327

[CR5] Bixler, R. P., Coudert, M., Richter, S. M., Jones, J. M., Pulido, C. L., Akhavan, N., Bartos, M., Passalacqua, P., & Niyogi, D. (2022). Reflexive co-production for urban resilience: Guiding framework and experiences from Austin, Texas. *Frontiers in Sustainable Cities*, 178:1015630.

[CR6] Briggs, G. A. (1973). Diffusion estimation for small emissions. *Atmospheric Turbulence and Diffusion Laboratory,**965*, 83–145.

[CR7] Cardil, A., Monedero, S., Silva, C. A., & Ramirez, J. (2019). Adjusting the rate of spread of fire simulations in real-time. *Ecological Modelling,**395*, 39–44. 10.1016/j.ecolmodel.2019.01.017

[CR8] CDC. (2021). *PLACES: Local data for better health*. https://www.cdc.gov/places/10.5888/pcd19.210459 PMC9258452 35709356

[CR9] U.S. Census Bureau. (n.d.). *2015–2019 ACS 5-year estimates census tract data*. Retrieved October 13, 2023, from https://data.census.gov/

[CR10] Chesnokova, S. (2025). “Wildfire digital twin” of the Earth: OroraTech grabs €37M to scale satellite-powered fire intelligence — TFN. *Tech Funding News*. https://techfundingnews.com/wildfire-digital-twin-of-the-earth-ororatech-grabs-e37m-to-scale-satellite-powered-fire-intelligence/

[CR11] City of Austin. (n.d.). *City of Austin open data portal*. Retrieved October 13, 2023, from https://data.austintexas.gov/

[CR12] El Saddik, A. (2018). Digital twins: The convergence of multimedia technologies. *IEEE Multimedia,**25*(2), 87–92. 10.1109/MMUL.2018.023121167

[CR13] Fakhruddin, B., Kirsch-Wood, J., Niyogi, D., Guoqing, L., Murray, V., & Frolova, N. (2022). Harnessing risk-informed data for disaster and climate resilience. *Progress in Disaster Science,**16*, 100254.

[CR14] FCAMMS. (n.d.). VSmoke-Web. *Fire consortia for advanced modeling of meteorology and smoke*. Retrieved October 13, 2023, from https://weather.gfc.state.ga.us/GoogleVsmoke/vsmoke-Good2.html

[CR15] Cal Fire. (2025). *Palisades fire*. https://shorturl.at/TwU0H

[CR16] Fuller, A., Fan, Z., Day, C., & Barlow, C. (2020). Digital twin: Enabling technologies, challenges and open research. *IEEE Access,**8*, 108952–108971. 10.1109/ACCESS.2020.2998358

[CR17] Glaessgen, E., & Stargel, D. (2012). The digital twin paradigm for future NASA and US Air Force vehicles. *53rd AIAA/ASME/ASCE/AHS/ASC Structures, Structural Dynamics and Materials Conference 20th AIAA/ASME/AHS Adaptive Structures Conference 14th AIAA*, 1818.

[CR18] Gong, H., Su, D., Zeng, S., & Chen, X. (2025). Parallel simulation and prediction techniques for digital twins in urban underground spaces. *Automation in Construction,**175*, 106212.

[CR19] Gubala, T. (n.d.). *BlenderPy script*. Retrieved October 13, 2023, from https://github.com/TylerGubala/blenderpy

[CR20] Hall, S., & Evarts, B. (2022). *Fire loss in the United States during 2021*. National Fire Protection Association.

[CR21] Horsley, J. A., Broome, R. A., Johnston, F. H., Cope, M., & Morgan, G. G. (2018). Health burden associated with fire smoke in Sydney, 2001–2013. *The Medical Journal of Australia,**208*(7), 309–310. 10.5694/mja18.00032 29642818 10.5694/mja18.00032

[CR22] Huang, Y., Li, J., & Zheng, H. (2024). Modeling of wildfire digital twin: Research progress in detection, simulation, and prediction techniques. *Fire,**7*(11), 412.

[CR23] Isikdag, U., Underwood, J., & Aouad, G. (2008). An investigation into the applicability of building information models in geospatial environment in support of site selection and fire response management processes. *Advanced Engineering Informatics,**22*(4), 504–519. 10.1016/j.aei.2008.06.001

[CR24] Jeong, D.-Y., Baek, M.-S., Lim, T.-B., Kim, Y.-W., Kim, S.-H., Lee, Y.-T., Jung, W.-S., & Lee, I.-B. (2022). Digital twin: Technology evolution stages and implementation layers with technology elements. *IEEE Access,**10*, 52609–52620. 10.1109/ACCESS.2022.3174220

[CR25] Kaginalkar, A., Kumar, S., Gargava, P., Kharkar, N., & Niyogi, D. (2022). SmartAirQ: A big data governance framework for urban air quality management in smart cities. *Frontiers in Environmental Science*, 228:785129.

[CR26] Kaginalkar, A., Kumar, S., Gargava, P., & Niyogi, D. (2021). Review of urban computing in air quality management as smart city service: An integrated IoT, AI, and cloud technology perspective. *Urban Climate,**39*, 100972.

[CR27] Kamath, H. G., Singh, M., Magruder, L. A., Yang, Z.L., & Niyogi, D. (2022). *GLOBUS: GLObal building heights for Urban Studies*. arXiv Preprint arXiv:2205.12224. 10.1038/s41597-024-03719-w PMC11327349 39147835

[CR28] Kamath, H. G., Singh, M., Malviya, N., Martilli, A., He, L., Aliaga, D., He, C., Chen, F., Magruder, L. A., & Yang, Z.-L. (2024). GLObal building heights for urban studies (UT-GLOBUS) for city-and street-scale urban simulations: Development and first applications. *Scientific Data,**11*(1), 886. 39147835 10.1038/s41597-024-03719-w PMC11327349

[CR29] Kaur, M. J., Mishra, V. P., & Maheshwari, P. (2020). The convergence of digital twin, IoT, and machine learning: Transforming data into action. In M. Farsi, A. Daneshkhah, A. Hosseinian-Far, & H. Jahankhani (Eds.), *Digital twin technologies and smart cities* (pp. 3–17). Springer International Publishing. 10.1007/978-3-030-18732-3_1

[CR30] Kim, H., Kwon, J.W., Yun, S., & Kim, W.T. (2019). A novel wildfire digital-twin framework using interactive wildfire spread simulator. *Eleventh International Conference on Ubiquitous and Future Networks (ICUFN),**2019*, 636–638. 10.1109/ICUFN.2019.8806107

[CR31] Lavdas, L. G. (1996a). Improving control of smoke from prescribed fire using low visibility occurrence risk index. *Southern Journal of Applied Forestry,**20*(1), 10–14. 10.1093/sjaf/20.1.10

[CR32] Lavdas, L. G. (1996b). *Program VSMOKE-users manual*. Southern Research Station.

[CR33] Lewis, R. H., Jiao, J., Seong, K., Farahi, A., Navrátil, P., Casebeer, N., & Niyogi, D. (2024). Fire and smoke digital twin – A computational framework for modeling fire incident outcomes. *Computers, Environment and Urban Systems,**110*, 102093. 10.1016/j.compenvurbsys.2024.102093

[CR34] Li, J., & Hu, M. (2025). Mapping spatial inequity in urban fire service provision: A Moran’s I analysis of station pressure distribution. *ISPRS International Journal of Geo-Information,**14*(4), 164.

[CR35] Li, X., Liu, H., Wang, W., Zheng, Y., Lv, H., & Lv, Z. (2022). Big data analysis of the Internet of Things in the digital twins of smart city based on deep learning. *Future Generation Computer Systems,**128*, 167–177. 10.1016/j.future.2021.10.006

[CR36] Li, Y., Zhang, T., Ding, Y., Wadhwani, R., & Huang, X. (2024). Review and perspectives of digital twin systems for wildland fire management. *Journal of Forestry Research,**36*(1), 14. 10.1007/s11676-024-01810-x

[CR37] Lochhead, I., & Hedley, N. (2019). Mixed reality emergency management: Bringing virtual evacuation simulations into real-world built environments. *International Journal of Digital Earth,**12*(2), 190–208. 10.1080/17538947.2018.1425489

[CR38] Mallia, D. V., Kochanski, A. K., Kelly, K. E., Whitaker, R., Xing, W., Mitchell, L. E., Jacques, A., Farguell, A., Mandel, J., & Gaillardon, P.E. (2020). Evaluating wildfire smoke transport within a coupled fire-atmosphere model using a high-density observation network for an episodic smoke event along Utah’s Wasatch Front. *Journal of Geophysical Research: Atmospheres,**125*(20), e2020JD032712.

[CR39] Mandalapu, A., Seong, K., & Jiao, J. (2024). Evaluating urban fire vulnerability and accessibility to fire stations and hospitals in Austin, Texas. *PLoS Climate,**3*(7), e0000448.

[CR40] Mendi, A. F. (2022). A digital twin case study on automotive production line. *Sensors*. 10.3390/s22186963 10.3390/s22186963 PMC9506524 36146313

[CR41] Mendi, A. F., Erol, T., & Doğan, D. (2022). Digital Twin in the Military Field. *IEEE Internet Computing*, *26*(5), 33–40. 10.1109/MIC.2021.3055153. IEEE Internet Computing

[CR42] Mohammadi, N., & Taylor, J. E. (2017). *Smart city digital twins*. pp.1–5.

[CR43] MSN. (n.d.). *Microsoft air quality map*. Retrieved October 13, 2023, from https://www.msn.com/en-in/weather/maps/airquality/in-undefined?zoom=15

[CR44] National Weather Service. (n.d.). *NWS Austin/San Antonio*. Retrieved October 13, 2023, from https://www.weather.gov/ewx/

[CR45] Neirotti, P., De Marco, A., Cagliano, A. C., Mangano, G., & Scorrano, F. (2014). Current trends in smart city initiatives: Some stylised facts. *Cities,**38*, 25–36. 10.1016/j.cities.2013.12.010

[CR46] Nguyen, T. H., Hung, N. T., Nagashima, T., Lam, Y. F., Doan, Q.-V., Kurokawa, J., Chatani, S., Derdouri, A., Cheewaphongphan, P., & Khan, A. (2022). Development of current and future high-resolution gridded emission inventory of anthropogenic air pollutants for urban air quality studies in Hanoi, Vietnam. *Urban Climate,**46*, 101334.

[CR47] NOAA Research. (n.d.). *The High-Resolution Rapid Refresh (HRRR)*. Retrieved October 13, 2023, from rapidrefresh.noaa.gov/hrrr

[CR48] Occhipinti, C., Aneja, V. P., Showers, W., & Niyogi, D. (2008). Back-trajectory analysis and source-receptor relationships: Particulate matter and nitrogen isotopic composition in rainwater. *Journal of the Air & Waste Management Association,**58*(9), 1215–1222. 18817114 10.3155/1047-3289.58.9.1215

[CR49] OpenTopography. (n.d.). *OpenTopography*. Retrieved October 13, 2023, from https://opentopography.org/

[CR50] Over, M., Schilling, A., Neubauer, S., & Zipf, A. (2010). Generating web-based 3D city models from OpenStreetMap: The current situation in Germany. *Computers, Environment and Urban Systems,**34*(6), 496–507.

[CR51] Pan, H., Dou, Z., Cai, Y., Li, W., Lei, X., & Han, D. (2020). Digital twin and its application in power system. *2020 5th International Conference on Power and Renewable Energy (ICPRE)*, 21–26. 10.1109/ICPRE51194.2020.9233278

[CR52] PurpleAir. (n.d.). *PurpleAir real-time air quality monitoring*. Retrieved October 13, 2023, from https://map.purpleair.com/

[CR53] Razavi, H., Titidezh, O., Asgary, A., & Bonakdari, H. (2024). Building resilient smart cities: The role of digital twins and generative AI in disaster management strategy. In S. Pourroostaei Ardakani & A. Cheshmehzangi (Eds.), *Digital twin computing for urban intelligence* (pp. 95–118). Springer Nature Singapore. 10.1007/978-981-97-8483-7_5

[CR54] Sadrabadi, M. T., Peiró, J., Innocente, M. S., & Rein, G. (2025). Conceptual design of a wildfire emergency response system empowered by swarms of unmanned aerial vehicles. *International Journal of Disaster Risk Reduction*. 10.1016/j.ijdrr.2025.105493

[CR55] NASA Science. (2024). *"Wildfire digital twin” pioneers new AI models and streaming data techniques for forecasting fire and smoke*. https://science.nasa.gov/science-research/science-enabling-technology/nasa-wildfire-digital-twin-pioneers-new-ai-models-and-streaming-data-techniques-for-forecasting-fire-and-smoke/

[CR56] Shie, R.H., & Chan, C.C. (2013). Tracking hazardous air pollutants from a refinery fire by applying on-line and off-line air monitoring and back trajectory modeling. *Journal of Hazardous Materials,**261*, 72–82. 23912073 10.1016/j.jhazmat.2013.07.017

[CR57] Shuman, J. K., Balch, J. K., Barnes, R. T., Higuera, P. E., Roos, C. I., Schwilk, D. W., Stavros, E. N., Banerjee, T., Bela, M. M., Bendix, J., Bertolino, S., Bililign, S., Bladon, K. D., Brando, P., Breidenthal, R. E., Buma, B., Calhoun, D., Carvalho, L. M. V., Cattau, M. E., … Zhang, X. (2022). Reimagine fire science for the anthropocene. *PNAS Nexus,**1*(3), pgac115. 10.1093/pnasnexus/pgac115 10.1093/pnasnexus/pgac115 PMC9896919 36741468

[CR58] Tashakkori, H., Rajabifard, A., & Kalantari, M. (2015). A new 3D indoor/outdoor spatial model for indoor emergency response facilitation. *Building and Environment,**89*, 170–182. 10.1016/j.buildenv.2015.02.036

[CR59] Turner, D. B. (2020). *Workbook of atmospheric dispersion estimates: An introduction to dispersion modeling, second edition* (2nd ed.). CRC Press. 10.1201/9780138733704

[CR60] Vrabič, R., Erkoyuncu, J. A., Butala, P., & Roy, R. (2018). Digital twins: Understanding the added value of integrated models for through-life engineering services. *Procedia Manufacturing,**16*, 139–146. 10.1016/j.promfg.2018.10.167

[CR61] Walker, B., Holling, C. S., Carpenter, S. R., & Kinzig, A. (2004). Resilience, adaptability and transformability in social–ecological systems. *Ecology and Society,**9*(2). 10.5751/ES-00650-090205

[CR62] White, G., Zink, A., Codecá, L., & Clarke, S. (2021). A digital twin smart city for citizen feedback. *Cities,**110*, 103064. 10.1016/j.cities.2020.103064

[CR63] Yan, J., Lu, Q., Li, N., Chen, L., & Pitt, M. (2025a). Common data environment for digital twins from building to city levels. *Automation in Construction,**174*, 106131.

[CR64] Yan, J., Lu, Q., Li, N., & Pitt, M. (2025b). Developing data requirements for city-level digital twins: Stakeholder perspective. *Journal of Management in Engineering,**41*(2), 04024068. 10.1061/JMENEA.MEENG-6434

[CR65] Yu, D., & He, Z. (2022). Digital twin-driven intelligence disaster prevention and mitigation for infrastructure: Advances, challenges, and opportunities. *Natural Hazards,**112*(1), 1–36. 10.1007/s11069-021-05190-x 35125651 10.1007/s11069-021-05190-x PMC8801275

[CR66] Zheng, X., Petrali, P., Lu, J., Turrin, C., & Kiritsis, D. (2022). RMPFQ: A quality-oriented knowledge modelling method for manufacturing systems towards cognitive digital twins. *Frontiers in Manufacturing Technology,**2*. 10.3389/fmtec.2022.901364

[CR67] Zheng, Y., Yang, S., & Cheng, H. (2019). An application framework of digital twin and its case study. *Journal of Ambient Intelligence and Humanized Computing,**10*(3), 1141–1153. 10.1007/s12652-018-0911-3

[CR68] Zohdi, T. I. (2020). A machine-learning framework for rapid adaptive digital-twin based fire-propagation simulation in complex environments. *Computer Methods in Applied Mechanics and Engineering,**363*, 112907.

[CR69] Zohdi, T. I. (2021). A digital twin framework for machine learning optimization of aerial fire fighting and pilot safety. *Computer Methods in Applied Mechanics and Engineering,**373*, 113446.

